# REDOX physical-chemical method boosted phospholytic bacteria technology for enhanced phosphorus solubilization

**DOI:** 10.3389/fbioe.2022.1124832

**Published:** 2023-01-04

**Authors:** Yongwei Jiang, Tao Cui, Lei Cao, Jian Huang, Yong Tu, Yong Chen, Yonghao Zhang, Anlin Xu, Junwei Zhou, Ming Ni, Kajia Wei

**Affiliations:** ^1^ Jiangsu Provincial Environmental Engineering Technology Co, Ltd., Nanjing, Jiangsu, China; ^2^ Jiangsu Province Engineering Research Center of Synergistic Control of Pollution and Carbon Emissions in Key Industries, Nanjing, China; ^3^ Jiangsu Province Engineering Research Center of Standardized Construction and Intelligent Management of Industrial Parks, Nanjing, China; ^4^ Key Laboratory of Jiangsu Province for Chemical Pollution Control and Resources Reuse, School of Environmental and Biological Engineering, Nanjing University of Science and Technology, Nanjing, China; ^5^ School of Environmental Science and Engineering, Yancheng Institute of Technology, Yancheng, China; ^6^ School of Environmental Science and Engineering, Nanjing Tech University, Nanjing, China

**Keywords:** redox process, phospholytic bacteria, phosphorus solubilization, electrochemical technology, resource recycle

## Introduction

Phosphate-solubilizing bacteria are the bacteria that secrete organic acids and phosphatases to convert the insoluble phosphorus fixed in soil into soluble phosphorus (such as monovalent H_2_PO_4_^−^ and divalent HPO_4_^2−^) (Rodriguez et al., 2006). Among the species, the main bacteria with phosphorus solubilizing ability were *Pseudomonas, Bacillus, Rhizobium, Burkholderia, Achromobacter, Agrobacterium, Microccocus, Erwinia, Aereobacter* and *Flavobacterium* (Ma et al., 2022). Representative phosphate-solubilizing bacteria are *Pseudomonas*, *Rhizobium* and *Bacillus*, which have the best phosphate-solubilizing capability (Maheshwari et al., 2013).

The mechanism of these bacteria can be explained based on the form of phosphorus, namely, mineral phosphorus and organic phosphorus. From the perspective of mineral phosphorus dissolution, the most accepted theory is that phosphate-solubilizing bacteria synthesize organic acids that acidify the cells of microorganisms and the surrounding environment, releasing phosphorus from mineral phosphorus through proton (H^+^) exchange of Fe^3+^, Ca^2+^, Al^3+^, etc., (Goldstein, 1994; Kim et al., 2005). Research has found gluconic acid to be the most effective organic acid for dissolving mineral phosphorus. It is generated from the action of phosphate-solubilizing bacteria such as *Pseudomonas sp.*, *Erwinia herbicola*, *Pseudomonas cepacian* and *Burkholderia cepacia*. Another representative organic acid is 2-ketogluconic acid, which is produced from the action of phosphate-solubilizing bacteria such as *Rhizobium leguminosarum*, *Rhizobium meliloti* and *Bacillus firmus*. In addition, there are also reports on other organic acids including glycolic acid, oxalic acid, malonic acid and succinic acid.

From the perspective of organic phosphorus dissolution, it is in its nature the mineralization of organic phosphorus. Saprophytes mineralize organophosphates when decomposing organic matter in soil, which releases orthophosphate and thus dissolves phosphorus (Illmer and Schinner, 1992). The mineralization of organic phosphorus is completed by the action of phosphatase, the main process of which is the hydrolysis of phosphate or phosphoric anhydride bonds. The function of phosphorus solubilizing bacteria is related to the type of phosphorus source itself. It has been confirmed that tricalcium phosphate and hydroxyapatite are more easily degraded than phosphates in inorganic phosphorus, while phospholipids and sugar phosphates are relatively easily decomposed and polyphosphates are more slowly decomposed (Adnane et al., 2021). In addition, the mineralization of organic phosphorus by bacteria is significantly influenced by environmental factors; for organic phosphorus, medium alkalinity is more favorable for its mineralization, unlike for inorganic phosphorus.

Although mechanisms for phospholytic bacteria technology has been discovered in a certain degree, several challenges are still remained in its practical employment. First, it usually causes a considerable delay for the phosphorous conversion at the early stages of phosphorus solubilization, when phosphorus-solubilizing bacteria have not yet formed large colonies or become dominant, such as in the initial stage of fertilization. Second, the accumulation of aromatic hydrocarbons and heterocyclic compounds (e.g., pesticide or herbicide) in the soil, which could not be utilized as the microbial carbon source, greatly inhibited the functioning of phospholytic bacteria. Moreover, the low temperature or the turbulence of environmental pH conditions also decreases the activity of bacteria, thus slowing down the phosphorus conversion rate. All these issues have great impacts on the practical applications of the phospholytic bacteria technology. Herein, seeking a facile but efficient technology to make a synergistic interaction with bio-tech for boosting of phosphorus solubilization efficiency is challenging but of great significance.

## Electrochemical enhancement of biological phosphorus solubilization

**FIGURE 1:**
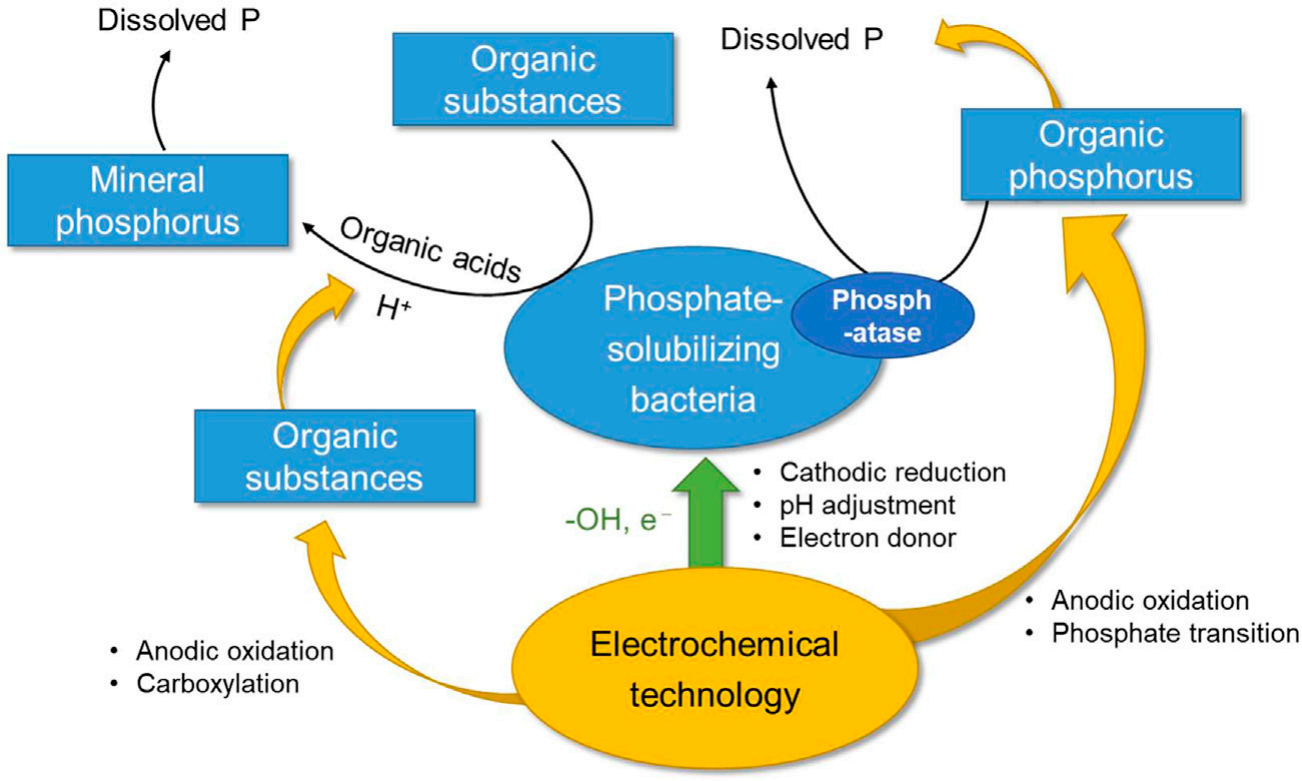
Schematic illustration of the electrochemical reduction-oxidation process on the phosphorus solubilization performance conducted by phospholytic bacteria technology.

Electrochemical technology is a kind of multi-functional physical-chemical process, containing electro-adsorption, electro-migration, anodic oxidation and cathodic reduction reactions (known as REDOX process), which plays an important role in the water and soil remediation. The high content of organic matter and a variety of electrolytes (such as the mineral salt and inorganic fertilizer) in soil and its wetness enabled electrochemical process to be realized in diverse ways. Electrochemical degradation of organic matter can be achieved directly by electron transfer between the electrode and the organic matter, or by the following reactions: at the anode with water molecules as the medium: H_2_O → **·**OH + H^+^ + e^−^, generating hydroxyl radical to oxidize the organic matter while lower the pH, and at the cathode: 2H^+^ + e^−^ = H_2_, consuming H^+^ to raise the pH (Wang et al., 2022). In general, electrochemistry can not only achieve the transformation of organic matter (such as conversing into low molecular acid or directly degrading the organophosphorus), but also alter ambient pH through the adjusting the above reactions on the anode and cathode, so as to adjust the soil pH according to the demand (Teklit et al., 2023). On the other hand, electrochemistry can stimulate the phosphate-solubilizing bacteria and enhance the activity of the bacteria, thus enhancing the metabolism of phospholytic bacteria (Huang et al., 2021; Liu et al., 2022). Combined with the phosphate-solubilizing mechanism of phosphate-solubilizing bacteria, both oxidation and reduction will to a certain extent enhance biological phosphorus solubilization. The illustration of the electrochemical reduction-oxidation process on the phosphorus solubilization performance conducted by phospholytic bacteria technology is referred to Figure 1.

## Phosphate solubilization of phosphate-solubilizing bacteria enhanced by anodic oxidation

Anodic oxidation is closely related to the properties of the electrode. Different oxygen evolution potential (OEP) of the anode will lead to different processes and endpoints of organic degradation. When OEP is less than 1.8V, the organic matter is degraded by the high-valence oxides generated from interaction between hydroxyl radical and anode rather than by hydroxyl radical, for which the degradation endpoint will be low molecule organic acids (such as formic acid and acetic acid) and other intermediates. Noble metal electrodes like ruthenium or ruthenium-iridium exhibit this property (Eleftheria et al., 2019). Therefore, introducing such electrodes to the phosphate-solubilizing bacteria system will enhance the content of organic acids and H^+^ in the system and thus improving inorganic phosphorus dissolution. In particular, a significant effect of anodic oxidation is that when soil contains some refractory organic matter, such as residual organic pollutants from pesticide spraying or fertilization, anodic oxidation can degrade such organic matter into low molecular acids, which can not only reduce soil toxicity and its harm to the flora (such as phosphorous solubilizing bacteria), but also improve the concentration of organic acid in the system (Brillas, 2021). On the other hand, extensive previous studies have confirmed that anodic oxidation could directly degrade organic phosphorus and convert it into soluble phosphate, thus enhancing the dissolution of organic phosphorus (Wang et al., 2022).

## Phosphate solubilization of phosphate-solubilizing bacteria enhanced by cathodic reduction

The mechanism of phosphate solubilization of phosphate-solubilizing bacteria enhanced by cathodic reduction is relatively simple—it is mainly the cathodic reduction that consumes H^+^ and transfer the pH of the system to neutral or alkaline (Cheng et al., 2021). As mentioned above, phosphate-solubilizing bacteria dissolve organic phosphorus through the hydrolysis of phosphate or phosphoric anhydride bonds by phosphatase, for which medium alkalinity is more favorable—fits right in with the cathode. Another important effect is that in an atmosphere with enough air, the cathode will react as follows: O_2_+2H^+^ + 2e^−^ = H_2_O_2_. The generated strong oxidizing substance, hydrogen peroxide (Brillas, 2022), promotes the conversion of organophosphorus to phosphoric acid roots, thus enhancing the dissolution of organophosphorus. In addition, it has been reported that cathode can not only reduce organic matter itself, but also enhance the reducing ability of bacteria. The electron giving ability of cathode can strengthen the electron transfer between cathode, microbiota and organic matter, and improve the reducing ability of the system (Yang et al., 2020). When there are nitro compounds in the system, cathodic reduction can achieve the enhanced removal of the substance and ensure the biological activity of phosphorus solubilizing bacteria (Hu et al., 2022).

## Future perspective

First of all, the high content of organic matter and a variety of electrolytes in soil and its wetness facilitate the introduction of electrochemical technology. Such characteristics of electrochemical technology as the oxidized organic matter staying in an organic acid state, producing H^+^ and H^+^ being consumed in reduction agree well with the phosphorus solubilization mechanism of phosphorus-solubilizing bacteria (Nancharaiah et al., 2016). Therefore, the introduction of electrochemical technology will more significantly improve the efficiency of phosphorus solubilization than a system with phosphorus-solubilizing bacteria alone. As stated above, anodic oxidation can enhance the dissolution of both inorganic phosphorus and organophosphorus, while cathodic reduction mainly enhances the dissolution of organophosphorus. In particular, the strengthening mechanism of anodic oxidation on phosphorus dissolution may play a positive role in promoting phosphorus circulation and even plant growth (Yang et al., 2021). Because the largest reserves of phosphorus in nature exist in the form of inorganic phosphorus, which is chelated into insoluble complexes such as Fe^3+^, Ca^2+^ and Al^3+^ in the soil. The content of soluble phosphorus in nature is very low, which greatly hinders the absorption and acquisition of plants (Wei et al., 2018). At the same time, electrochemical technology can not only create an environment suitable for the metabolism of phosphorus solubilizing bacteria, but also remove toxic organic matter in the surrounding environment which is not conducive to the growth of phosphorus solubilizing bacteria. On the other hand, the functioning of phosphorus solubilizing bacteria could be promoted under assistance of the REDOX process of the electrodes (Ren et al., 2022). Electrochemical enhanced phosphorus solubilization can be used in the early stages of phosphorus solubilization to ensure phosphorus conversion when phosphorus-solubilizing bacteria have not yet formed large colonies or become dominant; or after fertilization to enhance phosphorus solubilization when organic/inorganic phosphorus is not converted in time. The cathodic reduction could also maintain the medium alkalinity of the soil and provide electron to activate the metabolisms of the bacteria. As for the scale application, the challenges may be located at the distribution of the anode and cathode pair in the practical soil, the anti-corrosion of the electrode materials by the salt and bacteria and the cost control of the integrated electro-bacteria system. Overally, it is very promising to have the introduction of electrochemical technology into the phosphorus-solubilizing bacteria system, which is of great benefits for the transformation of phosphorus elements and plant growth (Rodríguez and Fraga, 1999; Acevedo et al., 2014; Wang and He, 2022).
